# Physical, Mechanical, and Morphological Properties of Hybrid *Cyrtostachys renda*/Kenaf Fiber Reinforced with Multi-Walled Carbon Nanotubes (MWCNT)-Phenolic Composites

**DOI:** 10.3390/polym13193448

**Published:** 2021-10-08

**Authors:** Tamil Moli Loganathan, Mohamed Thariq Hameed Sultan, Mohammad Jawaid, Qumrul Ahsan, Jesuarockiam Naveen, Ain Umaira Md Shah, Abd. Rahim Abu Talib, Adi Azriff Basri

**Affiliations:** 1 Department of Aerospace Engineering, Faculty of Engineering, Universiti Putra Malaysia, UPM Serdang 43400, Selangor Darul Ehsan, Malaysia; tamilmoli@yahoo.com (T.M.L.); abdrahim@upm.edu.my (A.R.A.T.); adi.azriff@gmail.com (A.A.B.); 2 Laboratory of Biocomposite Technology, Institute of Tropical Forestry and Forest Products (INTROP), Universiti Putra Malaysia, UPM Serdang 43400, Selangor Darul Ehsan, Malaysia; 3 Aerospace Malaysia Innovation Centre (944751-A), Prime Minister’s Department, MIGHT Partnership Hub, Jalan Impact, Cyberjaya 63000, Selangor Darul Ehsan, Malaysia; jawaid@upm.edu.my; 4 Department of Mechanical and Production Engineering, Ahsanullah University of Science and Technology (AUST), Dhaka 1208, Bangladesh; qumrul.mpe@aust.edu; 5 School of Mechanical Engineering, Vellore Institute of Technology, Vellore 632014, India; naveen.j@vit.ac.in

**Keywords:** *Cyrtostachys renda* fiber, kenaf fiber, multi-walled carbon nanotubes, phenolic, physical, mechanical properties, TOPSIS

## Abstract

Adequate awareness of sustainable materials and eco-legislation have inspired researchers to identify alternative sustainable and green composites for synthetic fiber-reinforced polymer composites in the automotive and aircraft industries. This research focused on investigating the physical, mechanical, and morphological properties of different hybrid *Cyrtostachys renda* (CR)/kenaf fiber (K) (10C:0K, 7C:3K, 5C:5K, 3C:7K, 0C:10K) reinforced with 0.5 wt% MWCNT–phenolic composites. We incorporated 0.5 wt% of MWCNT into phenolic resin (powder) using a ball milling process for 25 h to achieve homogeneous distribution. The results revealed that CR fiber composites showed higher voids content (12.23%) than pure kenaf fiber composites (6.57%). CR fiber phenolic composite was more stable to the swelling tendency, resulting in the lowest percentage of swelling rate (4.11%) compared to kenaf composite (5.29%). The addition of kenaf fiber into CR composites had improved the tensile, flexural, and impact properties. The highest tensile and flexural properties were found for weight fraction of CR and kenaf fiber at 5C:5K (47.96 MPa) and 3C:7K (90.89 MPa) composites, respectively. In contrast, the highest impact properties were obtained for 0C:10K composites (9.56 kJ/m2). Based on the FE-SEM image, the CR fiber lumen was larger in comparison to kenaf fiber. The lumen of CR fiber was attributed to higher void and water absorption, lower mechanical properties compared to kenaf fiber. 5C:5K composite was selected as an optimal hybrid composite, based on the TOPSIS method. This hybrid composite can be used as an interior component (non-load-bearing structures) in the aviation and automotive sectors.

## 1. Introduction

Green composites are generated from sustainable materials that provide wider opportunities to industries, the environment and end-customers as petroleum resources are harmful to the ecosystem. In the automotive and aviation industries, the transition towards more sustainable material is an initiative for a more viable ecosystem, cost-efficiency, and demand for European legislation [1]. The proposed fibers possess unique characteristics compared to synthetic fibers, such as being renewable, sustainable, having low environmental impact, abundant availability, lightweight, low cost, low density, non-toxic, good thermal stability, environmentally-friendly, low abrasion, as well as bio-degradable. Natural fiber’s ability to absorb carbon dioxide has a promising value in reducing environmental pollution. The cellulosic structure of natural fiber presents features analogous to synthetic fibers. These characteristics obstruct the ability of natural fibers to provide good reinforcement of polymeric composites.

*Cyrtostachys renda* (CR) is a plant that has a physical similarity to Areca catechu from the palm family. CR is an alternative plant fiber used to reinforce polymer composites as an added value to agro-waste materials. In the current analysis, this plant was chosen for its adaptability, abundance, and fast growth rates, which facilitate access to broad supplies of raw materials. This plant is typically cultivated for landscaping purposes, and its waste is underused. A previous study found that CR fiber is compatible with phenolic by improving its mechanical properties [2]. 

Kenaf fiber, known as *Hibiscus cannabinus* L. family Malvacea, is a type of bast fiber. It has great features of superior toughness, strength, and stiffness, and sufficient ability to strengthen polymers with a high aspect ratio relative to other fibers [3]. Kenaf is an economic crop that is not only grown in Malaysia but also in Mississipi, Texas, Florida, and North Carolina in the United States of America [4]. Reinforcement with kenaf is explored extensively in engineering applications, able to commercialize our nation’s natural fibers to the global market and eventually increase our economic growth. Kenaf fibers are natural resources that also lead to the production of environmentally safe and renewable assets for the automobile, manufacturing, recreation, food packaging and furniture industries [5,6], but not extensively to the aviation industries.

Wang et al. studied abutilon-fiber-reinforced poly(lactic acid) and found that the natural fiber of abutilon has outstanding energy absorbing properties, making it suitable for industrial applications [7]. A few researchers had carried out on hybridization of palm oil fiber and kenaf [8,9] and embarked on the mechanical properties of the composites. Hanan et al. studied the mechanical properties of hybrid composites by reinforcing the oil palm empty fruit bunch (EFB) and kenaf fiber mats with epoxy matrix [8]. They found that the tensile and flexural properties increased dramatically by increasing the amount of kenaf fiber to oil palm EFB composites. In contrast, the impact properties of pure EFB composites were far higher than those of hybrid composites. Selection of kenaf bast fiber was due to high tensile strength, while oil palm EFB was due to high toughness; these features compensate each property of bio-composite. Conclusively, the flexural stability of the bio-composite improves when the kenaf is hybridized with the reinforcement of palm fiber. Based on previous researchers’ findings, palm and kenaf combined into fiber compensate each other in tensile and impact properties to boost the mechanical properties of a hybrid composite.

In this study, bio-phenolic derived from Cashew Nut Shell Liquid (CNSL) is a renewable, low-cost, and bio-based matrix for aircraft applications. The cost of phenol varies between USD 1100–1450, while the cost of CNSL varies between USD 200–300 per metric tonne, depending on the processing and the content added. The fire-resistance rating recorded in various studies and reviews concluded that phenolic > polyimide > epoxy > polyester and vinyl ester [10]. Char forming is the path to high flame retardation and good fire efficiency. This allows phenolic resin desirable for structural applications, but phenolic resins have many drawbacks, including low strength and moisture development by condensation polymerization.

To meet specific requirements for a particular application, it is imperative to find advanced material with improved mechanical and multifunctional behavior. Nano filler-modified polymer matrix was considered an effective and versatile way to boost the mechanical features of fiber-reinforced polymer composites [11]. Among these fillers, multi-walled carbon nanotubes (MWCNT) have superior mechanical properties, excellent specific strength, thermal conductivity, and electrical conductivity compared to other carbon compounds [12,13]. Therefore, the use of MWCNT as reinforcement in polymer composites has attracted tremendous attention with new structural concepts in the composite field [14] with its extraordinary features. Eslami et al. found that the flexural strength and thermal stability of phenolic composites improve with the increase in MWCNT up to 0.5 wt% and decreases at 2 wt% [15]. In addition, Chaiwan and Pumchusak found that 0.5 wt% of MWCNT reinforced phenolic resin provided the highest mechanical properties in flexural and tensile strength using a dry dispersion method [16]. Therefore, the low content of MWCNT as inorganic nanoparticles is used as a modifier to toughen the phenolic composites. As lignocellulosic fiber is used as a reinforcement in the phenolic matrix [17,18], the adhesion between fiber and matrix is improved compared to other thermosets. Moreover, there is some interaction between the polar fiber groups and the phenolic matrix [19,20].

There are few studies have been carried out on MWCNT–phenolic and natural fiber reinforced phenolic composites, respectively. However, no work has been performed with MWCNT and hybrid natural fiber reinforced phenolic composites to date. The rationale behind the choice of these two fibers of CR and kenaf is high modulus (kenaf) and low modulus fiber (CR) can take the advantage of both constituents and improve the damage tolerance. In addition, CR from the palm family anticipated in providing high toughness compared to kenaf fiber. The aim of this study is to investigate the effect of hybridization of CR/kenaf reinforced MWCNT–phenolic composites. This research focused on investigating the physical, mechanical, and morphological properties of different hybrid CR/K (10C:0K, 7C:3K, 5C:5K, 3C:7K, 0C:10K) reinforced with 0.5 wt% MWCNT–phenolic composites. This research had also been devoted to producing hybrid bio-composite with natural fibers (CR and kenaf) as fillers and sustainable material for the transportation industry.

## 2. Materials and Methods

### 2.1. Materials

The physical and mechanical properties of CR and kenaf are shown in Table 1. Sodium Hydroxide (NaOH) pellet with a molecular weight of 40 g/mol and a density of 2.13 g/cm^3^ was purchased from R&M Chemicals, Chandigarh, India. Meanwhile, CR fiber was collected from Telok Panglima Garang, Malaysia plantation and extracted manually from the leaf stalk by retting process. Kenaf and MWCNT were purchased from ZKK Sdn Bhd, Selangor, Malaysia. MWCNT was synthesized using a chemical vapor deposition (CVD) technique consisting of 8 to 15 nanotube layers with a diameter and size range between 12 to 15 nm and 3 to 15 μm, respectively, with a purity of 97%. Chemovate Girinagar, Bangalore, India, supplied the phenolic resin (Novolac type) derived from CNSL mixed with a 10% hexamine hardener.

### 2.2. Preparation of CR and Kenaf Fiber

Dry CR fiber and K were ground until pulverized and sieved with a size of <0.3 mm using a sieve shaker machine. The fibers were then immersed distinctly in NaOH solution at a concentration of 3 wt% for an hour. Then, they were washed several times with distilled water to eradicate the residue and dried for 24 h in an air circulation oven with a temperature of 80 °C.

### 2.3. Fabricating of Composites

**Figure 1:**
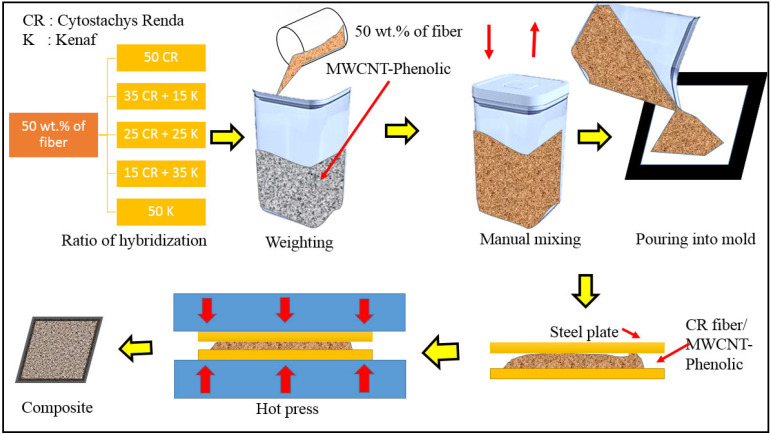
Schematic diagram of hybrid composite preparation.

0.5 wt% of MWCNT was added to the phenolic resin and dry mixed using a ball milling for 25 h to produce a homogeneous distribution. CR and/or kenaf hybrid composites with a composition shown in Table 2 were produced using a mold size of 150 mm × 150 mm × 3 mm. The fiber was uniformly mixed manually, with 0.5 wt% MWCNT–phenolic resin for 15 min. The mixture was then adequately spread into the mold. The molding plate was mounted in a hot press mold with a temperature of 150 °C and pressure of 10 tonnes, as illustrated in Figure 1. The composites were compressed for 10 min and were cold-pressed for 5 min.

## 3. Characterization

### 3.1. Physical Properties

#### 3.1.1. Density and Void Content

The density of the hybrid CR/K fiber-reinforced phenolic composites was determined using ASTM D1895. The density of each sample was determined using the following equation:

(1) $$\mathrm{Density}\left(g/cm^{3}\right)=\frac{\mathrm{Mass},\;m}{\mathrm{Volume},\;v}$$

where *m* denotes the mass and *v* denotes volume of the composite.

The void content of hybrid fiber-reinforced phenolic composites was calculated based on Equations (2) and (3), in accordance with ASTM D2734 method.

(2) $$\mathrm{Void}\;\mathrm{content}\left(\%\right)=\frac{\rho_{\mathrm{theoretical}}-\rho_{\mathrm{experimental}}}{\rho_{\mathrm{theoretical}}}\times 100$$

(3) $$\rho_{\mathrm{theoretical}}=\frac{1}{\left(\frac{W_{f}}{\rho_{f}}+\frac{W_{m}}{\rho_{m}}\right)}$$

where $W_{f}$ and $W_{m}$ denote the weight of fiber and matrix fraction in the composite, while $\rho_{f}$ and $\rho_{m}$ represent the density of fiber and matrix, respectively.

#### 3.1.2. Water Absorption and Thickness Swelling

CR and/or kenaf fiber hybrid reinforced with phenolic composite was examined for water absorption following ASTM D570. Five replicates were prepared for each set with a dimension of 10 mm × 10 mm × 3 mm. Prior to the testing, all samples were dried in an oven at 80 °C for 24 h and kept in a desiccator. The samples were weighed before immersion in distilled water at room temperature. After immersion for 24 h, samples were wiped and were weighed again. Weighing of the sample at 24 h immersion interval was conducted until achieving the saturation level. The percentage of water absorption was calculated using Equation (4):

(4) $$\mathrm{Water}\;\mathrm{absorption}\;\left(\%\right)=\frac{W_{1}-W_{0}}{W_{0}}\times 100$$

where, $W_{1}$ denotes the weight after immersion and $W_{0}$ denotes the weight before immersion.

The thickness swelling was determined for the samples that were used for the water absorption test. The initial thickness of the sample before immersion was measured. After immersion for 24 h in the water, the sample was wiped, and the thicknesses were measured again. The thickness measurement was also carried out for every 24 h interval until it reached the saturation level. The thickness swelling of the sample was determined by using Equation (5):

(5) $$\mathrm{Thickness}\;\mathrm{swelling}\;\left(\%\right)=\frac{T_{1}-T_{0}}{T_{0}}\times 100$$

where, $T_{0}$ denotes the original thickness of the sample and $T_{1}$ denotes the thickness after sample immersed for 24 h.

### 3.2. Mechanical Properties

#### 3.2.1. Tensile

The tensile test of C and K hybrid composites with a relative weight ratio of C:K fibers were carried out using an INSTRON 5556 (Instron, Norwood, MA, USA) according to ASTM D 3039 standard. Sample sizes of 120 mm × 20 mm × 3 mm were prepared. The head displacement and gauge length of the sample were maintained at 2 mm/min and 50 mm, respectively. The tensile strength and modulus values conform to the average of 5 replicates were recorded.

#### 3.2.2. Morphology

Tensile fracture surface morphology was performed using the Nova NanoSEM 230 Series Field Emission Scanning Electron Microscope (FESEM) (FEI, Hillsboro, OR, USA) to determine the dispersion and aggregation of CR and/or K in modified phenolic composites. The working distance was set between 5.2 mm and 6.3 mm, with an acceleration voltage of 10 keV. All the fractured specimens were sputter-coated with platinum to avoid charging effect for clear visualization.

#### 3.2.3. Flexural

The flexural properties of the composite samples were determined using three-point bending test in compliance with ASTM D 7264 standard using a universal testing machine INSTRON 5556 (Instron, Norwood, MA, USA). The sample dimension of 127 mm × 12.7 mm × 3.2 mm was prepared with the standard span to thickness ratio of 16:1. The crosshead speed was maintained at 2 mm/min, with a load cell of 5 kN. The flexural strength and modulus values correspond to the average of 5 replicates were recorded.

#### 3.2.4. Impact

The impact toughness of the composites was investigated using Izod impact testing. It was carried out according to ASTM D 256 at room temperature using a GT-7045-MD (Gotech, Taichung city, Taiwan) testing machine. The sample was prepared with dimensions of 64 mm × 12.7 mm × 3 mm. The energy that was absorbed during the impact testing is the amount of energy required to fracture the specimen completely. The impact strength of 5 replicates of each configuration was calculated based on the impact energy and the cross-sectional area of the sample, as shown in Equation (6).

(6) $$\mathrm{Impact}\;\mathrm{strength}=\frac{\mathrm{Impact}\;\mathrm{energy}\;\left(\mathrm{kJ}\right)}{\mathrm{Area}\;\left(m^{2}\right)}$$

### 3.3. Technique for Order Preference by Similarity to the Ideal Solution (TOPSIS)

The technique for Order Preference by Similarity to the Ideal Solution (TOPSIS) method is one of the (Multi-Criteria Decision Making) MCDM methods. It has been used in order to find an optimal hybrid ratio between CR and K reinforced with MWCNT–phenolic composites. The TOPSIS method considers both degrees of the distance of each alternative from positive and negative ideals, respectively. In this study, five criteria and five alternatives are ranked based on the TOPSIS method, as shown in Table 3. The following table describes the criteria and the weightage (based on importance) for the selection made according to the composite application.

There are few steps to be followed in order to obtain relative closeness. The steps are as follows:

Step 1: Normalize the decision-matrix

The normalized value *n_ij_* is obtained using Equation (7)

(7) $$r_{ij}\left(x\right)=\frac{x_{ij}}{\sqrt{\sum_{i=1}^{m}x_{ij}^{2}}}\;\;\;\;\;\;i=1,\ldots ,\;m\;\;\;;j=1,\;\ldots ,\;\;n$$

Step 2: Weighted normalized decision matrix

The normalized matrix is multiplied by the weight of the criteria, which is obtained by using Equation (8).

(8) $$v_{ij}\left(x\right)=w_{j}r_{ij}\left(x\right)\;\;\;\;\;\;\;i=1,\ldots ,\;m\;\;\;;j=1,\;\ldots ,\;n$$

Step 3: Determine the positive ideal and negative ideal solutions

The positive and negative ideal solutions are determined according to the following Equations (9) and (10).

(9) $$A^{+}=\left(v_{1}^{+},v_{2}^{+},\;\ldots ,\;v_{n}^{+}\right)\;A^{-}=\left(v_{1}^{-},v_{2}^{-},\;\ldots ,\;v_{n}^{-+}\right)$$

so that

(10) $$v_{j}^{+}=\left\{\left(max\;v_{ij}\left(x\right)|\;j\epsilon j_{1}),\;(min\;v_{ij}\left(x\right)|\;j\epsilon j_{2}\right)\right\}\;\;i=1,\ldots ,\;m\;v_{j}^{-}=\left\{\left(min\;v_{ij}\left(x\right)|\;j\epsilon j_{1}),\;(max\;v_{ij}\left(x\right)|\;j\epsilon j_{2}\right)\right\}\;\;i=1,\ldots ,\;m$$

where *j*_1_ and *j*_2_ denote the negative and positive criteria, respectively.

Step 4: Distance from the positive and negative ideal solutions

TOPSIS method rates each alternative based on the relative closeness degree to the positive ideal and distance from the negative ideal. Therefore, in this step, the calculation of the distances between each alternative and the positive and negative ideal solutions is obtained by using Equation (11).

(11) $$d_{i}^{+}=\sqrt{\overset{n}{\underset{j=1}{\sum }}{\left[v_{ij}\left(x\right)-v_{j}^{+}\left(x\right)\right]}^{2}}\;\;\;,\;\;\;i=1,\;\ldots ,\;m\;d_{i}^{-}=\sqrt{\overset{n}{\underset{j=1}{\sum }}{\left[v_{ij}\left(x\right)-v_{j}^{-}\left(x\right)\right]}^{2}}\;\;\;,\;\;\;i=1,\;\ldots ,\;m$$

Step 5: Calculate the relative closeness degree of alternatives to the ideal solution

In this step, the relative closeness degree of each alternative to the ideal solution is obtained by the following Equation (12). If the relative closeness degree has a value near 1, it means that the alternative has a shorter distance from the positive ideal solution and a longer distance from the negative ideal solution.

(12) $$C_{i}=\frac{d_{i}^{-}}{\left(d_{i}^{+}+d_{i}^{-}\right)}\;\;\;\;,\;\;\;i=1,\;\ldots ,\;m$$

## 4. Results and Discussion

### 4.1. Physical Properties

#### 4.1.1. Density and Void Content of CR-K Reinforced Phenolic Composites

Table 4 shows the density and void content of hybrid phenolic composites. Apparently, CR fiber composites show a higher amount of voids (12.23%) relative to pure kenaf fiber composites (6.57%). The void content was also connected to the hollow structure and porous nature of both CR and K, where the powdered resin cannot penetrate the frame structure of the fibers during impregnation. Pure CR fiber from the palm family, based on experimental results, had more porosity than K. This observation is similar to [21], which highlighted that incompatibility between phenolic and palm fiber leads to a high degree of void creation.

An increase in the void content is another factor attributable to the inadequate wetting of the fiber by the matrix [22] during the production phase of the composite. In this step of processing, wetting depends on how well the matrix content wets the fibers. Deficient wetting due to moisture in the fiber [23] and powered foam of the matrix had restricted wettability. Poor wetting of the fibers can lead to gaps between the fiber and the final solidified composites [24]. In the final composite, these holes act as a defect or develop as voids that decrease the composite’s ultimate strength.

In addition, the CR fiber surface was attached to the impurity’s substance, such as silica body [2]. This observation is similar to [21], which revealed incompatibility between phenolic and palm fiber contributes to a high degree of void creation. The addition of kenaf fiber into CR fiber composites had reduced the voids and porosity content. The kenaf fiber is more compatible with phenolic resin; this indication is supported by a similar finding [23]. Based on the measured density, kenaf fiber phenolic composites were more compact and had denser characteristics relative to CR composites. The decline in void content enhanced the mechanical and physical properties of hybrid composites [25].

#### 4.1.2. Water Absorption and Thickness Swelling of CR-K Reinforced Phenolic Composites

**Figure 2:**
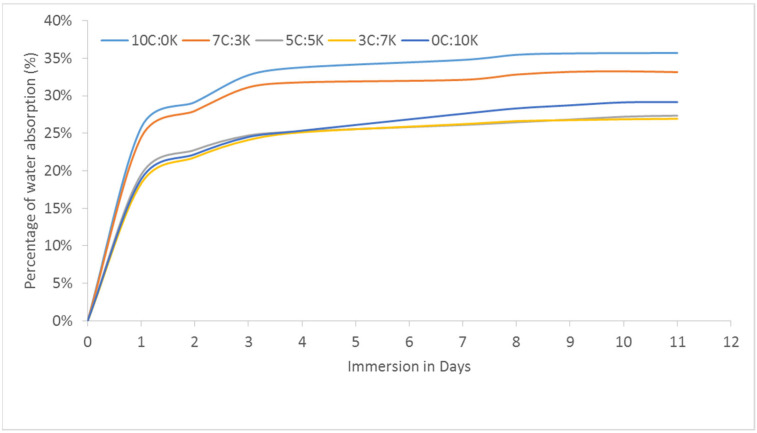
Water absorption percentage of CR and/or kenaf reinforced phenolic composites.

The water absorption percentage of CR and/or kenaf reinforced with phenolic composites curve was plotted against immersion days, as shown in Figure 2. From the graph, it is obvious that pure CR phenolic composites display high water absorption. Pure CR reinforced with phenolic composite was indicated to be more porous relative to kenaf fiber, which had permitted a significant amount of water molecules to diffuse to the composites. The hydrophilicity of the natural fiber depends on a number of hydroxyl groups responsible for absorbing water by the hydrogen bonding; this hydroxyl group is the key factor of water absorption and deterioration of the mechanical properties of the natural fiber-based composite [26,27]. These hydroxyl groups found in cellulose and hemicellulose offered quick access to water in the natural fiber-based composites. In natural fiber, there is an inter-fibrillary area of the cellulose framework that absorbs water molecules from the cracks and micro-voids that exist on the composite surface [28]. After being immersed for a long period in water, composites retained water molecules through capillary motion, and filled the composite voids and micro-cracks.

In contrast, pure kenaf fiber composites had been exposed to lower water absorption due to lower cellulose content [29] and lower porosity on the composite surface. Consequently, the compact properties of kenaf fiber enhanced the capillary mechanism to transport the holes and defects in the interface between the fiber and the phenolic matrix via the wettability phase. The analysis also showed that high water absorption of fiber was affected by the high fiber surface protection hole and the high exposed surface region [21]. It was also noticed that the addition of kenaf to CR fiber phenolic composite had caused a compact framework to reduce the gap and porosity of the surface exposed to composites. Therefore, it was observed that the water absorptions of 5C:5K, 3C:7K, and 0C:10K composites were identical to each other. The analysis also showed that fiber’s high water absorption was influenced by the high surface fiber protective gap and high surface exposure.

**Figure 3:**
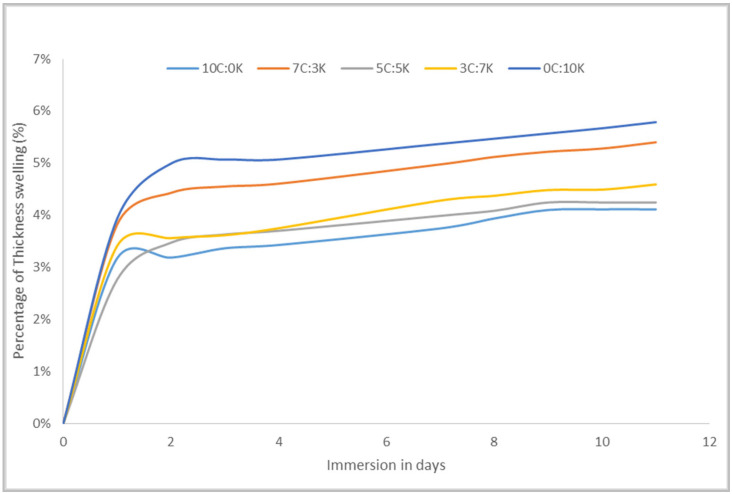
Thickness swelling percentage of CR and/or kenaf reinforced phenolic composites.

The thickness swelling percentage of CR and/or kenaf reinforced phenolic composites curve was plotted against immersion days, as shown in Figure 3. It can be seen that CR fiber phenolic composite was more stable to the swelling tendency, resulting in the lowest percentage (4.11%) of swelling, among others. Meanwhile, the highest percentage of thickness swelling resulted in kenaf-reinforced phenolic (6.29%) and hybrid composites thickness swelling falling between the thickness swelling of both pure fiber composites. Thickness swelling was closely related to the fiber composite dimensional stability, which was influenced by temperature and humidity [30]. The micro-cracks in the interfacial area induced by natural fiber swelling can promote the diffusion of water through micro-cracks and voids [26]. Weak interaction with fiber/matrix and fiber distribution influenced thickness swelling and dimensional stability of the composites [21]. It will restrict the swelling percentage and increase the dimensional stability of composites with a good interface and distribution between CR and kenaf fiber.

### 4.2. Mechanical Properties

#### 4.2.1. Tensile

**Figure 4:**
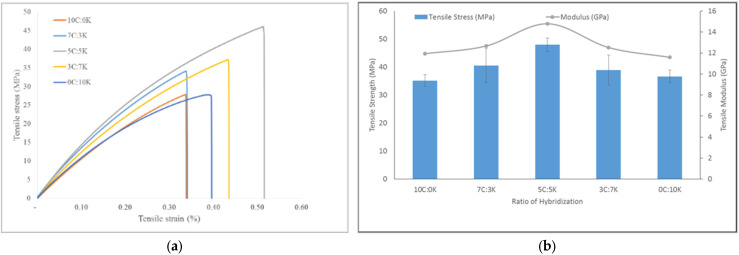
(**a**) Tensile stress vs. strain curve, (**b**) tensile strength and modulus, of hybridization between C and K fibers.

Figure 4a shows the graph of tensile stress vs. strain of C, K, and hybridization between C and K fiber at weight ration of 7C:3K, 5C:5K, and 3C:7K, respectively. It could be inferred that the addition of kenaf fiber in C composites had improved the tensile stress and strain in all cases. Moreover, hybrid composite with weight fraction of CR and kenaf fiber at 5C:5K ratio found the highest stress and stiffness properties.

Hanan et al. evaluated the tensile properties of the oil palm and kenaf fiber hybridization of epoxy enhanced composites and disclosed that the tensile and flexural properties were substantially improved by increasing the content of kenaf fiber to oil palm EFB composites [8]. The tensile strength of CR and kenaf composites obtained were 35.17 MPa and 36.67 MPa, respectively. Nevertheless, ultimate tensile strength was obtained for 5C:5K composite at 47.96 MPa. The hybridization of CR and kenaf fiber had enhanced the interfacial bonding between fibers and phenolic resin, thus promoted the tensile strength of the composites.

The tensile modulus of CR and kenaf was 11.94 GPa and 11.59 GPa, respectively. In comparison, hybridization of CR and kenaf fiber at a ratio of 5C:5K was enhanced to 14.79 GPa, as a positive hybrid effect. The addition of kenaf to CR phenolic composites increased the tensile modulus, which represented the ability of both fibers and matrix materials to conduct elastic deformation in small strains without interfacing composite fractures [31]. Furthermore, the weight fraction of kenaf in the CR composite increased, showing that tensile strength and modulus increased significantly. The incorporation of kenaf fiber into CR composites resulted in increases in the tensile strength and modulus up to 5C:5K; the further addition slightly decreased the tensile properties. The hybridization of CR and kenaf fiber increased the ability of the hybrid composites to withstand load and thus strengthened the hybrid composite’s stiffness. The incorporation of kenaf in composite composites was favorable for the mechanical behavior of hybrid composites because of the inherently strong mechanical characteristics of kenaf [8,32] over CR fiber. However, the poor adhesion between CR fiber and resin had caused the properties of tensile strength and modulus to be lower. Therefore, the reinforcement with other materials may improve their tensile performance. Furthermore, during the fabrication of composites, the void and small gap produced had probably induced inadequate wettability between the fiber and the matrix.

Table 5 shows the effect of hybridization of natural fibers phenolic composites on tensile properties. Interestingly, all the researchers used the same phenolic resin and studied on hybridization effect in terms of tensile properties in the same proportion. It was found that the combination of CR/kenaf at 5:5 ratio had almost provided a similar range of tensile strength with other natural fiber as found by [23]. However, 6% NaOH treated kenaf was more compatible with phenolic resin and enhancement in their tensile properties in comparison with 3% NaOH-treated kenaf. This fact is supported by [33], who found that 6% NaOH treated kenaf provided the highest tensile strength and modulus compared to 3% and 9% concentration, which may improve the fiber-matrix interaction. Interestingly, 3% NaOH-treated kenaf and CR provided more stiffness to the phenolic resin with the presence of 0.5 wt% of MWCNT. However, the combination of PALF-kenaf and CR-kenaf had provided positive hybrid effect than OP-SB, as investigated by [21].

#### 4.2.2. Morphology

**Figure 5:**
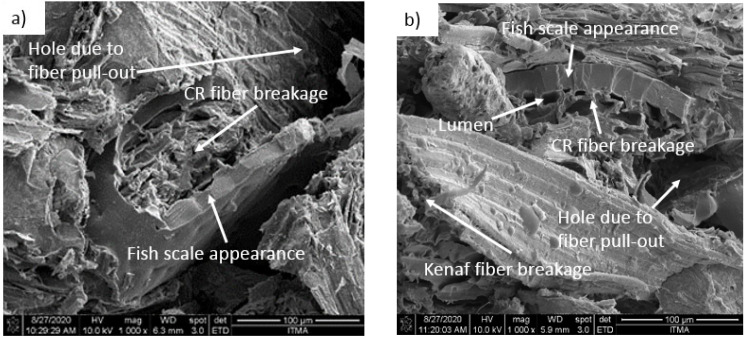

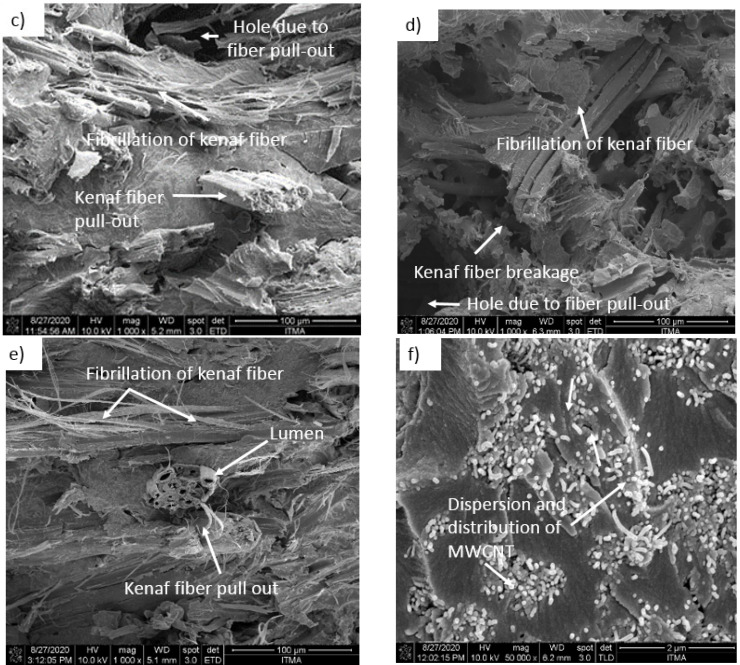
FE-SEM images of tensile fractured specimens of (**a**) 10C:0K, (**b**) 7C:3K, (**c**) 5C:5K, (**d**) 3C:7K (**e**) 0C:10K composites at magnification of ×1000, (**f**) 10C:0K composites at magnification of ×50,000.

A fractographic investigation was carried out using FESEM for a tensile fractured specimen. In order to examine the morphology and probable interface adhesion between matrix and fibers, micrographs taken at 1000× magnification were used. Figure 5a indicates that the fish scale appearance revealed the fiber adhered to the phenolic matrix and showed a good interface between CR fiber and MWCNT–phenolic resin. Meanwhile, Figure 5b–d demonstrate CR and kenaf hybrid fibers in the phenolic resin. It is noted that CR fiber lumen was larger in comparison to kenaf fiber. CR pore size had large openings, while kenaf had smaller pores that were compact in nature. It could be the reason the density of CR is lower than that of kenaf. From the figures, the CR fiber surfaces are rough and more porous (due to the removal of silica in certain cases), contributing to a decrease in density. This result affected the composite density, where the higher void content had a low density. The lumen of CR fiber influenced higher void and water absorption compared to kenaf fiber. However, the thickness of the swelling of the CR was lower than that of the kenaf due to the presence of the thick layer of the fish scale on the surface of the CR fiber, which resisted the dimensional change even the CR fiber absorbed the water.

**Figure 6:**
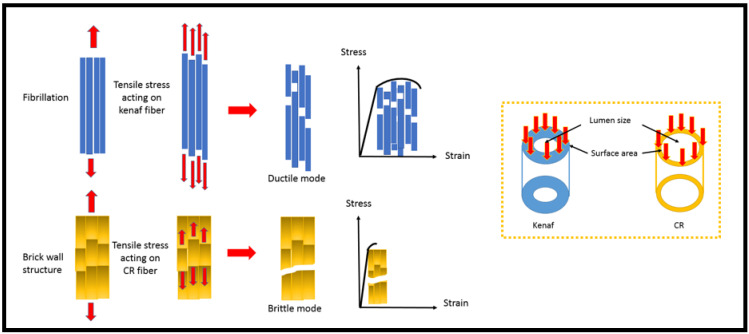
Schematic illustration of tensile fracture on CR and kenaf fiber.

Figure 5d,e clearly show the fibrillation of elementary fiber in the kenaf fiber bundle when the tension load was applied. Fibrillation occurred due to the removal of binding substances. It was confirmed by [35,36], who found that the alkalization mediated fiber fibrillation, such as the axial separation of elementary fibers had led to decreased fiber diameter, an increase in aspect ratio, and an efficient surface region. The elimination of surface impurities such as wax and cuticle in the surface on natural fibers had enhanced fiber–matrix adhesion by allowing both mechanical interlocking and bonding reactions. It also indicated that in the phenolic resin, the kenaf fiber failure was in ductile behavior mode, while the failure of CR fiber was in brittle behavior mode (Figure 5). The brick wall structure of CR fiber did not support the tensile load as much as the kenaf fiber. Another reason could be that the kenaf fiber was a denser and thicker wall (because of the smaller lumen size) than the CR fiber, so the load could be transferred more effectively, as illustrated in Figure 6. Fibril kenaf fiber prolonged the failure when the tensile load was applied, but there was a sudden fracture in the CR fiber when the tensile load was applied. Fibrils are more capable of rearranging themselves in the direction of tensile deformation, facilitating load distribution in the fibers and reducing the stress concentration [37]. As a result, kenaf fiber had been shown to improve ductility in MWCNT–phenolic by as much as 25% compared to the CR fiber.

#### 4.2.3. Flexural

**Figure 7:**
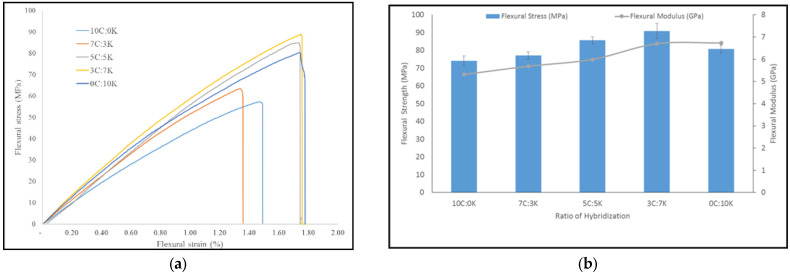
(**a**) Flexural stress vs. strain curve and (**b**) flexural strength and modulus, of hybridization between CR and K fibers.

Figure 7a,b show the flexural stress vs. strain curve, also flexural strength and modulus of C, K, and hybridization between C and K fiber at a weight ratio of 7C:3K, 5C:5K, and 3C:7K, respectively. The flexural strength of the CR composite obtained was 74.16 MPa, while the kenaf composite was 80.72 MPa. It was observed that the addition of kenaf fiber in CR reinforced composites had improved the flexural stress and strain in all cases. It could be inferred that kenaf fiber was more compatible with phenolic resin to enhance the flexural properties compared to CR fiber. The flexural strength and stiffness increased with the addition of kenaf fiber in CR composites; however, it showed maximum flexural strength (90.89 MPa) and stiffness (6.70 GPa), respectively, for hybrid composite at the proportion of 3C:7K. A similar finding was reported by [8], who found that the highest flexural strength was obtained at the proportion of 3:7 of oil palm: kenaf fiber, reinforced composites. It was noticed that the flexural strength and modulus were the lowest for CR composites. CR fiber is basically the palm fiber family and has similar physical and mechanical properties as oil palm fiber as reported by [2]. Therefore, it is reasonable to be compared with oil palm fiber.

Researchers who worked with oil palm (OP) fiber, particularly in random orientation, reported that their ability to withstand stress is generally poor and that if the OP fiber proportion is increased, the effect will be intensified [38]. The same fact was also reported by [8,39], who found that EFB fiber in hybrid composites indicated a reduction in flexural properties when their proportions were increased in hybridization with other natural fiber. Yusoff et al. reported that the tensile and flexural properties had showed a decreasing trend as the OP fiber loading was increased [40]. The mixture of CR and kenaf fibers had shown an increase in the flexural properties of MWCNT–phenolic composites as both fibers exhibit a good distribution in phenolic resins. Thus, excellent adhesion properties between two fibers of one matrix phase were effectively accomplished, thereby impacting the efficiency of the transition of load between the matrix and the reinforcement.

#### 4.2.4. Impact

**Figure 8:**
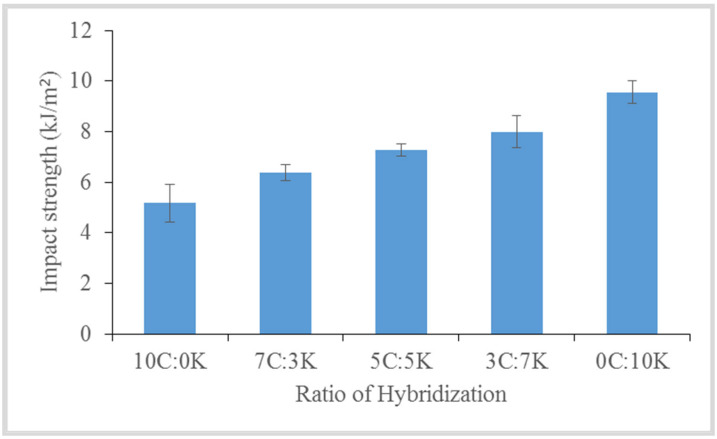
Impact strength of hybridization between C and K fibers.

The impact strength of hybrid CR/kenaf fiber reinforced with MWCNT–phenolic composites is shown in Figure 8. It was observed that the 0C:10K composite exhibited the highest impact strength of 9.56 kJ/m^2^, whereas the 10C:0K composites provided the lowest impact reading of 5.17 kJ/m^2^ compared to other composites. It was also observed that the addition of kenaf fiber into CR composites had increased the impact strength compared to the CR phenolic composites. Kenaf fiber is more compatible with the phenolic matrix in order to enhance the impact strength. A similar result was found by [41], where the addition of kenaf fiber had improved the impact strength of novolac phenolic resin from 4.77 kJ/m^2^ to 7.8 kJ/m^2^. Improvement of impact strength was attributed to the existence of crystalline cellulose in high crystalline kenaf fibers [42] and fibrillation. In phenolic composites, NaOH-treated kenaf fiber fibrillation dissipated more fracture energy and thus increased toughness [43]. The results imply that kenaf fiber complements CR fiber-reinforced phenolic composites to improve the impact properties. Furthermore, the chemical structure and architecture of the kenaf and CR fiber’s cell wall (primary wall, secondary wall, and middle lamella) provided higher impact properties for phenolic composites. Pristine phenolic has very poor impact power relative to fiber-reinforced polymer composites [44]. It is apparent that fiber plays an important role in the composite’s impact strength as it interacts with a crack forming inside the matrix and serves as a stress transfer medium.

### 4.3. TOPSIS Method

The best properties of hybrid composite for potential applications had been selected and ranked by the TOPSIS method. The decision matrix, normalization matrix, weight-normalized matrix, positive and negative ideal values, distance to positive and negative ideal points, relative closeness value known as ci value and ranking are shown in Table 6, Table 7, Table 8, Table 9 and Table 10.

The decision matrix is shown in Table 6, while the normalized matrix is represented in Table 7. Table 8 and Table 9 show the weighted normalized decision matrix and the positive and negative ideal values, respectively. Table 10 tabulates the distance to the positive and negative ideal solutions and the relative closeness degree of each alternative to the ideal solution.

**Figure 9:**
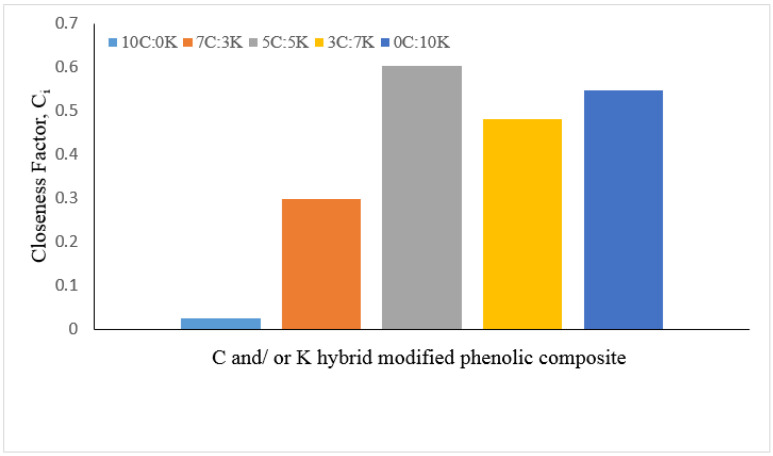
Relative closeness factor of C and/or K hybrid MWCNT–phenolic composite.

Finally, the hybrid composite rating is shown in Figure 9, relying on their proportion and properties. The results showed that the 5C:5K composite obtained the relative closeness to the ideal solution, and the values were 0.603, which was selected as the best hybrid proportion between CR and kenaf fiber phenolic composite. It was observed that the ranking of composite materials is as follows: Rank 1 (5C:5K), Rank 2 (0C:10K), Rank 3 (3C:7K), Rank 4 (7C:3K), and Rank 5 (10C:0K). The TOPSIS approach was the best method for choosing the optimal proportion of hybrid composites from the composite range [45].

## 5. Conclusions

CR fiber composites showed a higher amount of voids content and lower thickness swelling relative to pure kenaf fiber composites. The addition of kenaf fiber in CR composites had improved the tensile stress, tensile strain, flexural stress, flexural strain, and impact strength in all cases. The highest tensile stress, stiffness properties, flexural stress, strain, and impact properties were found for weight fraction of CR and kenaf fiber at 5C:5K, 3C:7K, and 0C:10K composites. Based on the FE-SEM images, CR fiber lumen is larger than kenaf fiber, and kenaf fiber failure is in ductile behavior mode, while the failure of CR fiber is in brittle behavior mode. The bigger lumen size of CR fiber influences higher void and water absorption compared to kenaf fiber. The 5C:5K proportion obtained was the relative proximity to the optimal solution and was chosen as the best hybrid proportion between CR and kenaf fiber reinforced with phenolic composites based on the TOPSIS method. Even though, this CR/kenaf reinforced MWCNT–phenolic composites showed higher mechanical properties, it is lower than the synthetic fiber-based composites. Therefore, it is recommended to investigate the effect of different nano-filler compatible with phenolic resin or hybrid synthetic/natural fiber based phenolic composites on the mechanical performance.

## Figures and Tables

**Table 1:** Physical and mechanical properties of CR and kenaf fiber.

Properties	CR	K
Density (g/cm^3^)	1.4	1.5
Tensile strength (MPa)	119	223–930
Tensile Modulus (GPa)	1.5	11–53
Elongation at break (%)	3.13	1.6–10
Cellulose (%)	45.42	31–39
Hemicellulose (%)	18.97	8–13
Lignin	20.70	12.1

CR: *Cyrtostachys renda*; K: Kenaf.

**Table 2:** Formulation of hybrid composites.

Designation Ratio of Hybrid Composites	0.5 wt.% MWCNT + Phenolic Resin (wt%)	CR (wt%)	K (wt%)
10C:0K	50	50	0
7C:3K	50	35	15
5C:5K	50	25	25
3C:7K	50	15	35
0C:10K	50	0	50

**Table 3:** Characteristics of Criteria.

Properties	Type	Weight
Density	−	0.1
Water absorption	−	0.1
Tensile	+	0.25
Flexural	+	0.25
Impact	+	0.3

**Table 4:** Density and void content of hybrid CR/K reinforced phenolic composites.

Composites	Theoretical Density (g/cm^3^)	Measured Density (g/cm^3^)	Void Content (%)
10C:0K	1.39	1.22	12.23%
7C:3K	1.39	1.26	9.35%
5C:5K	1.38	1.27	7.97%
3C:7K	1.37	1.27	7.30%
0C:10K	1.37	1.28	6.57%

**Table 5:** Hybridization of fibers phenolic composites on tensile properties.

Hybrid Fibers		Tensile Strength (MPa)					Tensile Modulus (GPa)					Ref.
50 wt.%		Proportion of Fiber 1:Fiber 2					Proportion of Fiber 1:Fiber 2					
Fiber 1	Fiber 2	10:0	7:3	5:5	3:7	0:10	10:0	7:3	5:5	3:7	0:10	
oil palm empty fruit bunch (OP)	sugarcane bagasse (SB)	4.95	5.56	5.23	5.34	4.51	0.58	0.66	0.63	0.64	0.52	[21]
pineapple leaf fiber (PALF)	6% NaOH treated kenaf	33.15	42.60	42.80	46.96	48.00	6.85	6.57	6.50	6.84	6.80	[23]
3% NaOH treated CR	3% NaOH treated Kenaf	35.17	40.55	47.96	38.91	36.67	11.94	12.67	14.79	12.52	11.59	Present study

Neat phenolic tensile strength = 22.25 MPa, Tensile Modulus = 4.15 GPa [34].

**Table 6:** Decision matrix.

	Density	Water Absorption	Tensile	Flexural	Impact
10C:0K	1.22	35.71	35.17	74.16	5.17
7C:3K	1.26	33.15	40.55	77.08	6.37
5C:5K	1.27	26.93	47.96	84.61	7.28
3C:7K	1.27	26.93	38.91	90.89	8.13
0C:10K	1.28	29.16	36.67	80.72	9.56

**Table 7:** The normalized matrix.

	Density	Water Absorption	Tensile	Flexural	Impact
10C:0K	0.430	0.617	0.363	0.354	0.367
7C:3K	0.448	0.447	0.461	0.376	0.396
5C:5K	0.451	0.363	0.600	0.489	0.414
3C:7K	0.451	0.363	0.399	0.526	0.455
0C:10K	0.455	0.394	0.369	0.467	0.574

**Table 8:** The weighted normalized matrix.

	Density	Water Absorption	Tensile	Flexural	Impact
10C:0K	0.043	0.062	0.091	0.088	0.110
7C:3K	0.045	0.045	0.115	0.094	0.119
5C:5K	0.045	0.036	0.150	0.122	0.124
3C:7K	0.045	0.036	0.100	0.131	0.137
0C:10K	0.045	0.039	0.092	0.117	0.172

**Table 9:** The positive and negative ideal values.

	Positive Ideal	Negative Ideal
Density	0.043	0.045
Water absorption	0.036	0.062
Tensile	0.150	0.091
Flexural	0.131	0.088
Impact	0.172	0.110

**Table 10:** Distance to positive, negative ideal points, and the relative closeness value and ranking.

Composites	Distance to Positive and Negative Ideal Points		The Relative Closeness Value and Ranking	
	Distance to Positive Ideal	Distance to Negative Ideal	Relative Closeness, C_i_	Rank
10C:0K	0.099	0.002	0.024	5
7C:3K	0.074	0.032	0.299	4
5C:5K	0.049	0.074	0.603	1
3C:7K	0.062	0.057	0.481	3
0C:10K	0.006	0.072	0.547	2
